# Supplementary material for: Improvement of Diet after an Early Nutritional Intervention in Pediatric Oncology
Source: Children (Basel). 2023 Mar 31;10(4):667. doi: 10.3390/children10040667 (PMC10137074; doi:10.3390/children10040667)
Supplement: Supplementary file 1 [file children-10-00667-s001.zip › children-2280450-Supplementary.pdf]

Table S1. Difference in the anthropometric characteristics of boys and girls between the initial assessment and after one year of nutritional intervention

Characteristics (z-scores)	Difference for boys (one year – initial)			Difference for girls (one year – initial)		
	n (total)	Mean z-score ± SD	*p*-value	*n* (total)	Mean z-score± SD	*p*-value
Weight	19	0.52 ± 0.65	0.003	17	0.03 ± 0.69	0.872
Height	19	-0.07 ± 0.56	0.610	17	-0.29 ± 0.52	0.032
BMI	19	0.74 ± 0.90	0.002	17	0.24 ± 0.80	0.228
Waist circumference	3	0.29	0.250	2	Too small	
MUAC	9	0.58 ± 1.27	0.004	4	-0.55	0.250
TSFT	7	1.03 ± 1.40	0.099	3	0.13	0.750
SSFT	5	0.05	0.625	2	Too small	
Systolic blood pressure	19	0.60	0.396	13	-0.52	0.236
Diastolic blood pressure	18	-0.25 ± 0.77	0.186	13	-0.06 ± 1.14	0.862

Anthropometric and clinical data were collected at initial visit and after one year of nutritional intervention. Data at initial assessment and after one year of nutritional follow-up were compared using paired t-tests or Wilcoxon. *p*-value <0.05 is considered statistically significant. BMI: body mass index [weight (kg) / height (m^2)]; MUAC: mid-upper arm circumference; TSFT: triceps skinfold thickness; SSFT: subscapular skinfold thickness; SD: standard deviation

Table S2. Difference in the biochemical data of boys and girls at the initial assessment and after one year of nutritional intervention

**Biochemical data**	**Difference for boys (one year – initial)**			**Difference for girls (one year – initial)**		
	n total	Mean difference ± SD	*p*-value	*n* total	Mean difference ± SD	*p*-value
HbA1c (%)	13	0.12 ± 0.86	0.636	9	-0.60±0.57	0.014
Vitamin D (mmol/L)	11	20.60 ± 36.69	0.092	10	7.84± 12.72	0.083
C-reactive protein (mmol/L)	11	0.60	0.898	11	-0.40	0.078
Total cholesterol (mmol/L)	13	0.27	0.377	12	0.03	0.895
HDL-C (mmol/L)	13	0.22 ± 0.39	0.058	11	0.32 ± 0.37	0.017
LDL-C (mmol/L)	13	-0.18	0.6848	11	-0.23 ±0.83	0.387
Non HDL-C (mmol/L)	13	0.20	0.542	11	-0.40 ± 1.02	0.222
Triglycerides (mmol/L)	13	-0.06	0.723	12	-0.15	0.110
0-9 years (mmol/L)	7	-0.46 ± 0.89	0.218	9	-0.40 ± 0.79	0.167
10-18 years (mmol/L)	5	0.37	0.500	3	Too small	

Non-fasting blood samples were collected during clinical visits. Data at initial assessment and after one year of nutritional intervention were compared using paired t-tests or Wilcoxon tests. *p*-value <0.05 is considered statistically significant. HbA1C: glycosylated hemoglobin; HDL-C: high-density lipoprotein cholesterol; LDL-C: low-density lipoprotein cholesterol; Non HDL-C: non high-density lipoprotein cholesterol; SD: standard deviation.
